# Correction: α-Synuclein modulates tau spreading in mouse brains

**DOI:** 10.1084/jem.2019219302262026c

**Published:** 2026-03-18

**Authors:** Fares Bassil, Emily S. Meymand, Hannah J. Brown, Hong Xu, Timothy O. Cox, Shankar Pattabhiraman, Chantal M. Maghames, Qihui Wu, Bin Zhang, John Q. Trojanowski, Virginia M.-Y. Lee

Vol. 218, No. 1 | https://doi.org/10.1084/jem.20192193 | October 22, 2020

The authors regret that, during figure preparation, several incorrect panels were placed in Fig. 5 and Fig. S2. In Fig. 5 C, the first two images partially overlapped (3 mpi WT and SynKO retrosplenial cortex images). In Fig. S2, the WT ADPHF entorhinal cortex image in panel A contained a partial overlap with the WT control sonication entorhinal cortex image in panel D, and the entorhinal cortex image in panel E overlapped with the 6 mpi MIX entorhinal cortex image in Fig. 3 B. These images have been replaced to remove the duplication. In addition, the following images in Fig. S2 have been replaced with new images from the corresponding mouse groups: 1) the WT MIX entorhinal cortex image in panel A; 2) the top SynKO ADPHF hippocampus image in panel B; 3) the SynKO ADPHF auditory cortex image in panel B; and 4) the SynKO ADPHF entorhinal cortex image in panel B.

The images carry no interpretive weight, and the figure legends and original conclusions of the article have not changed. The original and corrected Fig. 5 and Fig. S2 are shown here. The errors remain only in print and in PDFs downloaded before March 10, 2026.

**Figure:**
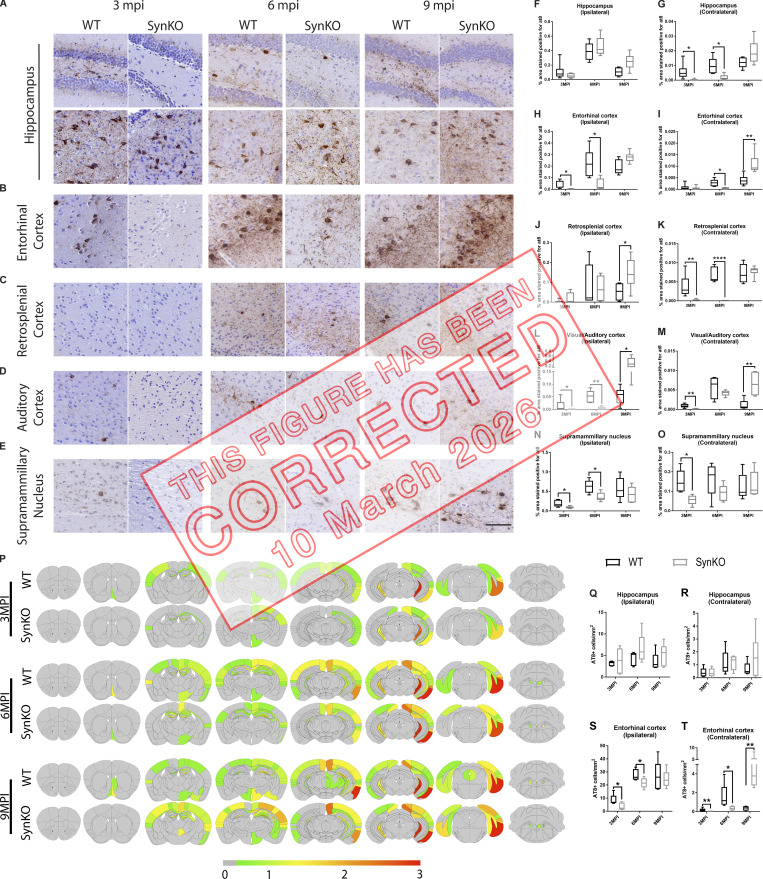


**Figure 5.**
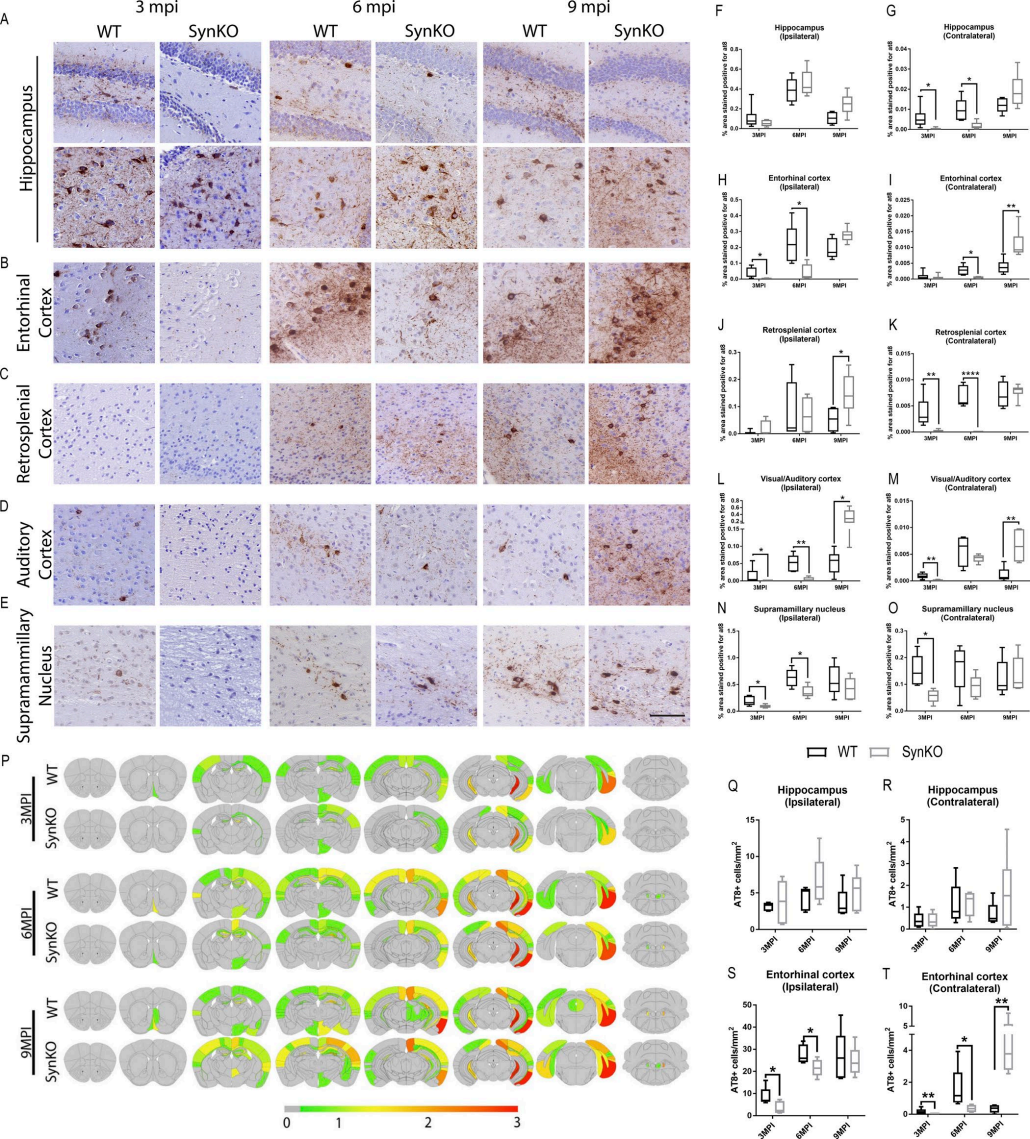
**α-Syn expression modulates pathology spread after AD-tau extract injection. (A–E)** Representative images for AT8 p-tau pathology in tissue sections from the rostral and caudal hippocampus (A), entorhinal cortex (B), retrosplenial cortex (C), auditory cortex (D), and supramammillary nucleus (E) of WT and α-synKO mice 3, 6, or 9 mpi of AD-tau–enriched extracts. Scale bar, 100 µm. **(F–O)** Quantification of p-tau seen in A–E in the ipsilateral (F) and contralateral (G) hippocampus, ipsilateral (H) and contralateral (I) entorhinal cortex, ipsilateral (J) and contralateral (K) retrosplenial cortex, ipsilateral (L) and contralateral (M) auditory cortex, and ipsilateral (N) and contralateral (O) supramammillary nucleus. A two-tailed *t* test was performed to calculate the difference between groups; *, P < 0.05; **, P < 0.01; ****, P < 0.0001. Data are presented as mean ± SEM (*n* = 5–9 mice per group). **(P)** Semiquantitative heat mapping of p-tau pathology in WT and α-synKO mice injected with human AD-tau–enriched extracts. **(Q–T)** Quantification of p-tau–positive neurons in the ipsilateral hippocampus (Q), contralateral hippocampus (R), ipsilateral entorhinal cortex (S), and contralateral entorhinal cortex (T) of WT and α-synKO mice injected with human AD-tau extract 3, 6, or 9 mpi. A two-tailed *t* test was performed to calculate the difference between groups; *, P < 0.05; **, P < 0.01. Data are presented as mean ± SEM (*n* = 5–6 mice per group). All experimental data were verified in at least two independent experiments.

**Figure:**
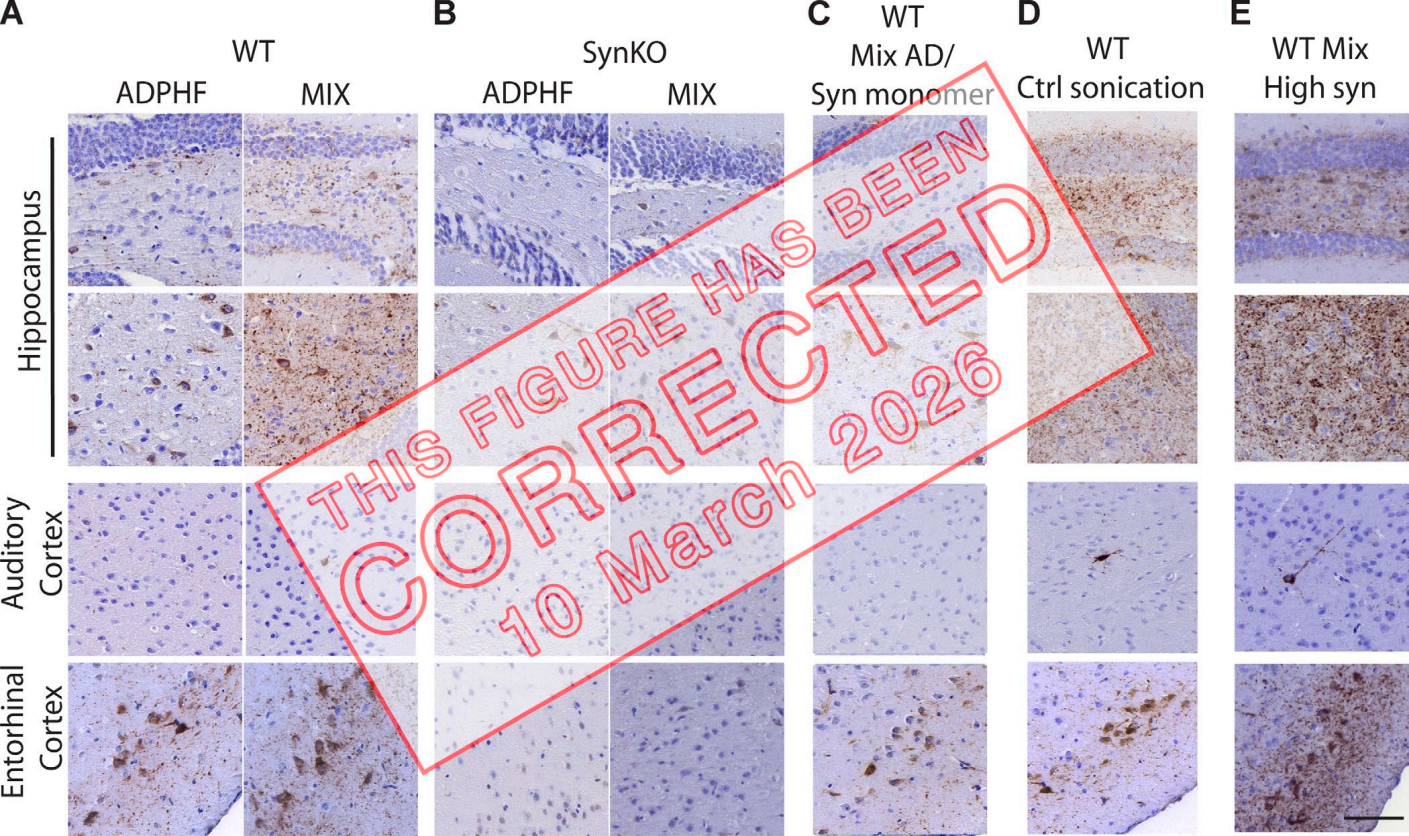


**Figure S2.**
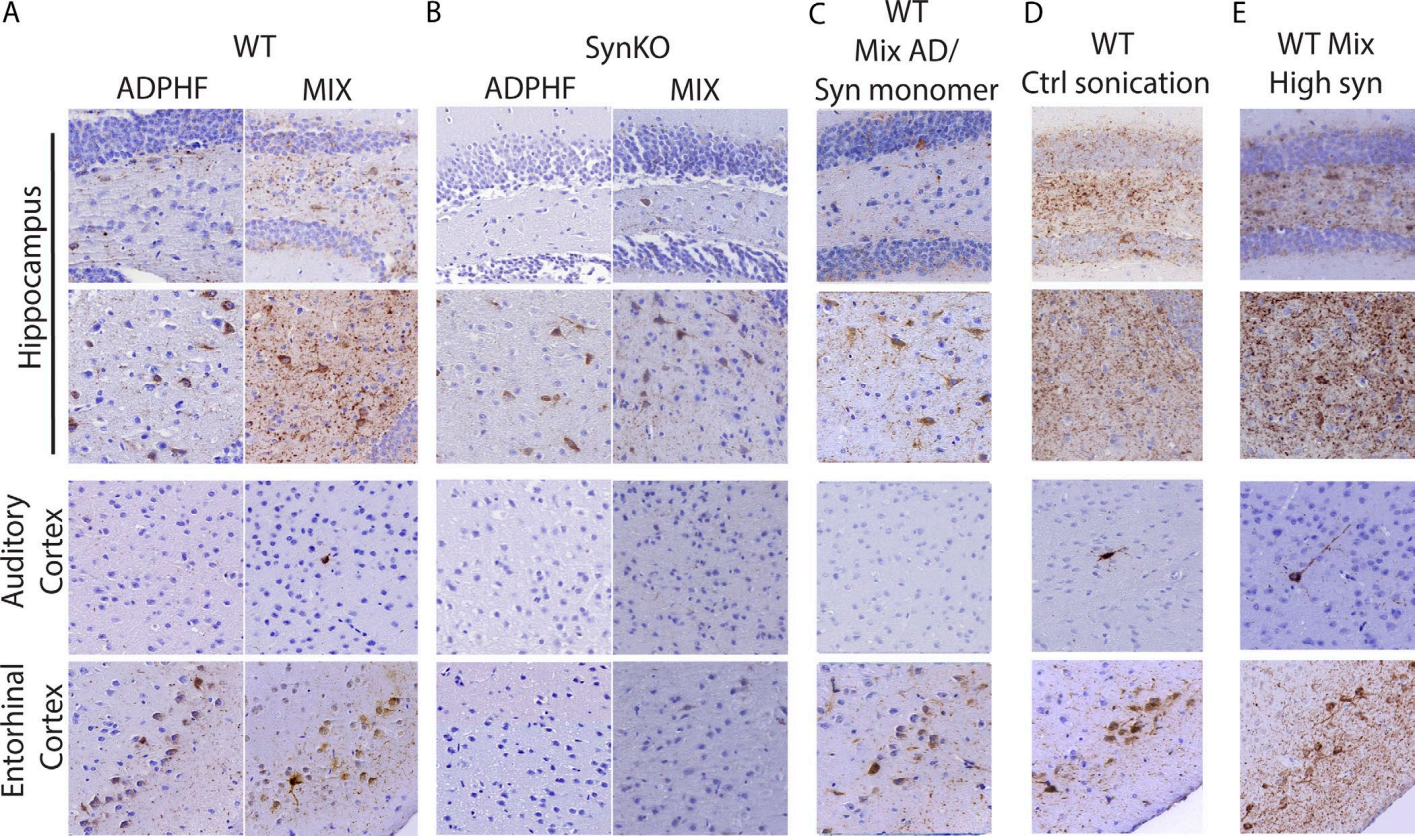
**Neuritic p-tau pathology is modulated by the presence of α-syn. (A–E)** P-tau staining 3 mpi in the hippocampus, auditory cortex, and entorhinal cortex (A) injected with either human AD-tau–enriched extracts alone or combined with α-syn mpffs (0.4 mg/ml) in WT and α-synKO mice, (B) mice injected with either human AD-tau–enriched extracts alone or combined with α-syn mpffs (0.4 mg/ml) as well as WT mice injected with human AD-tau–enriched extracts plus α-syn monomers (C), human AD-tau–enriched extracts, and α-syn mpffs injected after separate sonication (D), or human AD-tau–enriched extracts and a higher concentration of α-syn mpffs (2 mg/ml; E). Scale bar, 100 µm. All experimental data were verified in at least two independent experiments.

